# Temporary Anchorage Devices in Combined Treatments: Bridging Orthodontics and Prosthetics: Temporary Anchorage Devices in Combined Treatments

**DOI:** 10.31661/gmj.v13iSP1.3662

**Published:** 2024-12-08

**Authors:** Parastou Nastarin, Reza Sharifi, Mortaza Hosenzadegan, Mahya Gharouni, Maliheh Habibkhodaei

**Affiliations:** ^1^ Department of Orthodontics, Faculty of Dentistry, Tabriz University of Medical Sciences, Tabriz, Iran; ^2^ Department of Prosthodontics, Faculty of Dentistry, Tabriz University of Medical Sciences, Tabriz, Iran; ^3^ Department of Oral and Maxillofacial Radiology, Dental Faculty, Tabriz University of Medical Sciences, Tabriz, Iran; ^4^ Department of Prosthodontics, School of Dentistry, Alborz University of Medical Sciences, Karaj, Iran

**Keywords:** Temporary Anchorage Devices (TADs), Orthodontics, Prosthetics, Anchorage Devices, Combined Treatments, Biomechanics, Interdisciplinary Approach

## Abstract

Temporary Anchorage Devices (TADs) have become a cornerstone in modern orthodontics and prosthetic dentistry, offering reliable skeletal anchorage that enhances treatment precision and flexibility. This review explores the application of TADs in combined orthodontic-prosthetic treatments, emphasizing their growing significance in interdisciplinary care. This review aims to assess the role of TADs in addressing complex treatments that involve both orthodontic tooth movement and prosthetic restoration, while also examining their clinical effectiveness, treatment protocols, and anticipated innovations. TADs have revolutionized orthodontic treatments by providing stable anchorage for space closure, distalization, and intrusion, which would otherwise be challenging with traditional methods. They are increasingly utilized in prosthetic dentistry, particularly in cases involving edentulous spaces or insufficient bone support, where they provide temporary anchorage until permanent solutions, such as implants, can be placed. Several case studies demonstrate the effectiveness of TADs in aligning teeth and supporting prosthetic devices, highlighting their ability to streamline treatment and improve both functional and aesthetic outcomes. Technological advancements, such as 3D printing and digital planning tools, are improving the precision of TAD placement. Concurrently, innovations in biomaterials, like bioactive coatings, are enhancing osseointegration and reducing the risk of complications. As these technologies evolve, TADs are expected to become an integral part of fully digital treatment workflows, further improving outcomes in interdisciplinary cases. TADs serve as a critical tool in bridging orthodontics and prosthetics, providing enhanced control and versatility in complex treatments. Future research will likely focus on improving their design and expanding their applications, ensuring they remain a pivotal component of modern dental care.

## Introduction

Temporary Anchorage Devices (TADs) have revolutionized modern orthodontics and prosthetic treatments by providing a stable, non-mobile anchorage solution for various complex dental movements and reconstructions [1]. Historically, anchorage has been a central challenge in orthodontics, where unwanted tooth movements could compromise treatment outcomes [2]. Traditional methods, such as headgear or intraoral anchorage using adjacent teeth, often had limitations in terms of patient compliance, precision, and biomechanical control. In the late 1990s, the advent of TADs, small screws or mini-implants placed in the bone, marked a turning point by offering skeletal anchorage that minimized these challenges and provided more predictable results in both orthodontic and prosthetic cases [3].

TADs were originally developed for simpler orthodontic tasks, such as molar intrusion or retraction. However, their applications have since expanded considerably [4]. Today, they are widely used in complex orthodontic cases, such as open bite corrections and distalization, where traditional methods might fall short [5]. Also, these devices have seen growing applications in interdisciplinary treatments that integrate both orthodontics and prosthetics [6].

In prosthetic treatments, TADs act as temporary stabilizers during restorations and are particularly valuable in cases where conventional anchorage methods fail, such as in patients with significant tooth loss or compromised dental structures [7]. For example, recent studies have highlighted the use of these devices in edentulous patients as temporary supports for prosthetic appliances before permanent dental implants are placed, demonstrating their versatility beyond conventional orthodontics [8].

Growing interest in TADs as a bridging tool between orthodontics and prosthetics underscores the need for a comprehensive review. While orthodontic research has well-documented the applications of these devices in tooth movement, there is a relative paucity of literature exploring their combined use with prosthetic treatments [3]. As dental treatments become more interdisciplinary, understanding the synergistic potential of TADs is crucial for clinicians aiming to provide holistic and efficient patient care [5]. Complex cases that require both orthodontic alignment and subsequent prosthetic reconstruction, such as those involving significant tooth loss, asymmetry, or occlusal irregularities, can benefit immensely from the precise anchorage that these devices provide [1].

TADs represent a pivotal advancement in both orthodontic and prosthetic dentistry, providing stable skeletal anchorage for complex treatments (Schätzle, 2014) [2]. Traditionally, orthodontic treatments relied on intraoral or extraoral devices, which were often limited by precision and patient compliance [3]. These devices, however, provide a minimally invasive, temporary solution that enhances the predictability of tooth movements and prosthetic outcomes [9].

Thus, this review aims to address the gap in the literature by summarizing the role of TADs in combined orthodontic-prosthetic treatments. It will evaluate the clinical effectiveness of these devices, explore treatment protocols that integrate both specialties, and discuss potential future innovations. By doing so, this article will provide clinicians with valuable insights into the interdisciplinary use of TADs and help improve outcomes in complex dental treatments.

## 1. TADs: An Overview

TADs are small, screw-like devices designed to provide stable and fixed skeletal anchorage during orthodontic and prosthetic treatments [6]. Unlike traditional anchorage methods, which rely on teeth or external headgear, these devices are directly inserted into the bone, either in the maxilla or mandible, offering a more precise and reliable point of support [3]. This ability to provide skeletal anchorage without relying on dentition has significantly enhanced the range of orthodontic movements possible, making TADs indispensable in modern orthodontics and combined orthodontic-prosthetic treatments [9].

### 1.1.Definition and Types of TADs

TADs are generally classified based on their material composition, size, placement location, and intended function. Table-1 presents the popular classification of these devices. The two most common types of TADs are mini-implants and micro-screws [10]. Mini-implants are made primarily from biocompatible materials such as titanium, which offers a combination of strength and biocompatibility. These devices range from 6 to 12 mm in length and are typically used for larger anchorage tasks, such as distalization of molars or intrusion of entire dental arches [7].

Micro-screws, on the other hand, are smaller and often used in more delicate movements, such as individual tooth intrusion or space closure. These screws are typically placed in the interradicular spaces between teeth, avoiding vital structures such as nerves or roots, and are more flexible in placement due to their smaller size [11].

The placement location of TADs can vary based on the clinical need. Maxillary or mandibular insertion is common, and these devices can be placed in areas like the palate, alveolar bone, or even the zygomatic arch for specific cases [12]. The material used, typically titanium or titanium alloys, ensures that these devices integrate well with bone tissue but remain temporary by design, allowing for easy removal after treatment [1]. Some studies have also explored the use of bioactive coatings or alternative materials to enhance osseointegration and reduce the risk of infection or failure [6]. The primary function of TADs is to provide skeletal anchorage, which allows for controlled and predictable tooth movements without the need to rely on patient compliance with external devices or intraoral structures that may shift [3]. The biomechanical principle behind TADs is straightforward by anchoring directly to the bone, these devices create a stable point from which orthodontic forces can be applied. This skeletal anchorage minimizes unwanted side effects such as reciprocal movements that often occur when using conventional anchorage from adjacent teeth [13].

TADs work by allowing orthodontists to apply forces in any direction, whether pushing, pulling, or rotating, with the anchor point remaining stable [7]. For example, in cases of molar intrusion, these devices are inserted into the palate or posterior alveolar bone, and elastic chains or coils apply an upward force to intrude the molars [14]. Similarly, in distalization treatments, TADs placed in the maxilla allow for the retraction of anterior teeth or the entire arch without the need for headgear, greatly enhancing patient comfort and compliance [9].

The advantage of TADs lies in their ability to reduce the reliance on complex intraoral mechanics and patient-dependent factors [1]. This has led to their increasing use in both orthodontics and prosthetics, particularly in combined treatments where anchorage control is critical for both tooth movement and the stabilization of prosthetic appliances [6][14].

## 2. TADs in Orthodontics

**Figure-1:**
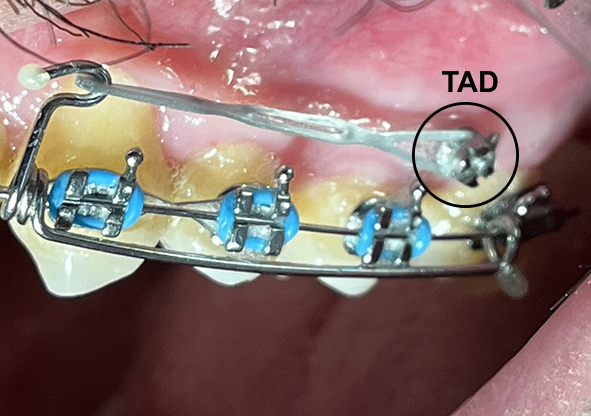
Figure 1. shows TAD placement in Orthodontic Treatment (distalization) in upper jaw of a man.

**Table:** Table1. Classification of TADs

**Type**	**Material**	**Placement Location **	**Function**	**Common Use**
Mini-screws	Titanium	Maxilla or Mandible	Skeletal Anchorage	Distalization
Micro-implants	Stainless Steel	Interradicular Spaces	Intrusion	Space Closure
Orthodontic Pins	Titanium Alloy	Palate	Anchorage for Intrusion	Correction of Open Bite

**Table:** Table2. Comparison of TADs in Orthodontics and Prosthetics

**Parameter**	**Orthodontics**	**Prosthetics**
Primary Objective	Provide temporary anchorage [24]	Stabilize prosthetic appliances [25]
Typical Applications	Space closure, intrusion, retraction [17]	Occlusal adjustment, edentulous spaces [26]
Average Duration of Use	6-12 months [20]	Varies based on case complexity (often less than 6 months) [27]
Success Rate	79-96% [28]	100% [29]
Common Placement Sites	Inter radicular spaces, palate, and posterior maxilla/mandible [30].	Edentulous ridges, particularly in areas lacking sufficient bone support for traditional implants [31]
Potential Complications	Soft tissue irritation, infection, or failure due to poor osseointegration or mechanical stress. Failure rates are higher in areas with thin cortical bone [32].	Infection, irritation, or early loosening, particularly in patients with low bone density or poor oral hygiene. May interfere with the placement of permanent implants if not managed carefully [31].
Advantages	Highly precise, reduces unwanted reciprocal tooth movement, and eliminates the need for patient-dependent devices (e.g., headgear) [15]	Offers a temporary, non-invasive anchorage solution before permanent implants; avoids the need for complex surgical procedures in compromised patients [33]
Limitations	Risk of failure in areas with low bone density; requires surgical placement and careful post-operative hygiene [17]	Short-term use, with risks of loosening or infection. Temporary solution requiring later permanent intervention (e.g., implants or fixed bridges) [34]

One of the most common applications of TADs in orthodontics is space closure, where they are used to facilitate the retraction of anterior teeth without relying on reciprocal forces from other teeth [12]. This is especially important in patients with missing teeth or in cases of significant crowding, where there is no available dentition to serve as an anchor [15]. TADs provide the stability needed to pull anterior teeth posteriorly, creating space or realigning the dental arches [16]. Recent research has shown that the use of these devices in space closure significantly reduces treatment time compared to conventional methods, improving the overall efficiency of orthodontic care [17]. Distalization is another key area where TADs have proved invaluable [18]. Traditional distalization techniques, such as headgear, are not only cumbersome but also heavily reliant on patient compliance [16]. With these devices, molars can be moved distally without relying on bulky external devices. For example, TADs can be placed in the posterior maxilla to retract the molars, creating space in the anterior region for the alignment of teeth [19]. Several studies have highlighted that the use of TADs for molar distalization results in less unwanted movement of adjacent teeth, enhancing the precision and success of the treatment [12]. In intrusion treatments, TADs are essential for correcting deep bites or extruded teeth [20]. Intruding molars or incisors require anchorage that can resist the vertical forces needed to push teeth into the bone [21]. These devices provide an optimal solution by anchoring in the maxilla or mandible and applying downward forces with precision [22]. For instance, in open bite cases, TADs inserted into the palate or alveolar bone allow for the vertical repositioning of molars, which in turn aids in the correction of occlusal planes [23]. Table-2 provides a comparison of TADs in both orthodontics and prosthetics.

### 2.1. Clinical Applications

TADs are indispensable in complex orthodontic procedures that require precise control over tooth movement. One notable clinical application is in asymmetrical tooth movements, where specific teeth need to be moved in different directions simultaneously [35]. Figure-1 shows TAD placement in Orthodontic Treatment (distalization) in upper jaw of a man.

For example, in cases of dental midline discrepancies, TADs can be placed to anchor the molars while allowing for the correction of the midline by applying targeted forces to the incisors [7]. Similarly, in open bite correction, these devices have been used to intrude on the posterior teeth, closing the open bite without affecting the position of the anterior teeth [36].

### 2.2. Success Rates and Complications

The success of TADs in orthodontic treatment is well-documented, however success rate was varied in different studies [28]. it was ranging between 79% and 96%, depending on the placement site, patient factors, and operator skill [28][37] (Table-2). Table-3 shows the important factors influencing the TAD Success rate. Research indicates that these devices exhibit particularly high success rates when placed in regions with dense cortical bone, such as the posterior maxilla or mandible [12]. Also, the minimally invasive nature of TAD placement allows for quicker recovery and a lower risk of complications compared to other orthodontic devices that require more invasive procedures [11]. However, despite their advantages, TADs are not without potential complications. One common issue is soft tissue irritation, especially when TADs are placed in areas with thin mucosa. This can lead to discomfort and inflammation around the insertion site [38]. In rare cases, TADs can also fail to osseointegrate, which results in their loosening or displacement. Failure rates tend to be higher in areas with low bone density, such as the anterior maxilla, where the bone may not provide adequate support for the device [39]. Poor oral hygiene can contribute to soft tissue infections around the TAD, leading to premature removal [40]. Recent advances in these device designs, including the use of bioactive coatings, have shown promise in reducing these risks by promoting better tissue healing and osseointegration [41].

## 3. TADs in Prosthetics

**Table:** Table3. Key Factors Influencing TAD Success

**Factor**	**Impact on TAD Success **
Bone Density	Higher success in dense bone
TAD Design	Thread design and material composition
Patient Compliance	Important for hygiene and stability
Surgical Technique	Precise insertion reduces failure rates

TADs have expanded their clinical utility beyond orthodontics and are increasingly used in prosthetic dentistry, particularly in situations where traditional prosthetic approaches are limited [42]. Their role in providing temporary anchorage for prosthetic treatments is especially valuable in challenging cases such as edentulous patients or when anatomical structures complicate conventional prosthetic solutions [43]. The application of TADs in prosthetics bridges the gap between orthodontics and prosthetic restoration, offering novel interdisciplinary treatment possibilities [21] (Table-2).

### 3.1. Clinical Applications

In prosthetic dentistry, TADs are primarily used in cases where conventional methods of anchorage are insufficient, such as in edentulous spaces or patients with severe bone resorption [44]. Traditional prosthetic treatments often rely on natural teeth or permanent implants to provide stability for prosthetic devices like dentures or bridges. However, in cases where these options are unavailable, TADs can serve as temporary anchors, offering a stable platform for supporting prosthetic appliances during the interim period before permanent solutions, such as osseointegrated implants, can be placed [7]. For example, in pre-implantation procedures, TADs can be used to temporarily support a dental prosthesis while the bone undergoes augmentation or healing after grafting [45]. Perez-Varela et al.[46] demonstrated that the use of these devices in treating skeletal Class III malocclusion is an effective alternative to surgery, particularly in adult patients. They highlighted the successful application of TADs for mandibular arch distalization, which, when coupled with skeletal anchorage and class III elastics, allowed for precise biomechanical control without significant negative effects on facial aesthetics or mandibular plane rotation. Similarly, Kim et al.[47] further reinforced that mandibular distalization using TADs reaches its anatomical limit at the root level of the second molar, an area where pushing beyond risks periodontal damage. Moreover, TADs are increasingly employed in implant-retained overdentures where immediate stabilization is needed but sufficient bone for permanent implants is lacking [48]. these devices provide support in areas where conventional implants might fail due to inadequate bone volume, particularly in the posterior mandible and maxilla. Their use allows for a more predictable outcome in maintaining proper alignment and retention of the prosthetic device until more definitive treatment can be performed [20]. In complex cases where both orthodontic movement and prosthetic restoration are required, TADs can be used to coordinate and enhance the outcomes of both specialties. Song et al.[12] examined the stability of total arch distalization in adult patients using these devices. Their research demonstrated that TADs provide reliable anchorage, leading to effective distalization of molars and incisors without the need for premolar extractions. Notably, they found that while some minor mesial drift occurred during the retention phase, the relapse in both maxillary and mandibular teeth was clinically insignificant, and soft tissue changes were stable throughout the observation period [12].

### 3.2. Success Rates and Complications

The use of TADs in prosthetic dentistry has gained considerable traction, particularly in complex cases involving edentulous spaces or compromised bone structures where traditional anchorage is inadequate [44]. While TADs are generally employed as a temporary solution before the placement of permanent implants, their success is influenced by several patient-specific and procedural factors [37]. The reported success rates of these devices in prosthetic applications typically up to 100% [29].

One of the key determinants of TAD success in prosthetic cases is bone quality. (Table-3) Research indicates that these devices are more stable and effective when inserted into areas with high-density cortical bone, typically found in the posterior mandible [49]. However, success rates decline when TADs are placed in regions with softer or thinner bone, such as the posterior maxilla, where bone resorption or atrophy is more common in edentulous patients [31]. This bone density variability means that pre-operative imaging and careful selection of the insertion site are crucial for ensuring TAD stability during the healing period of prosthetic treatments [50]. Despite their effectiveness, TADs are associated with complications that can compromise treatment outcomes. The most common issue is soft tissue irritation around the insertion site, particularly when these devices are placed in areas with thin mucosal coverage [51].

This irritation can lead to inflammation, discomfort, and, in some cases, soft tissue hypertrophy, which may necessitate the removal of the TAD before the prosthetic phase is complete [29]. Another frequent complication is infection, often linked to poor oral hygiene. The peri-implant tissues around TADs can become inflamed, leading to peri-implantitis or even the loosening of the device, which negatively impacts the prosthetic support [2]. Moreover, mechanical complications, such as early loosening of the TAD, can occur, especially in patients with low bone density or those subjected to excessive occlusal forces. This is particularly problematic in cases where these devices are providing interim support for prosthetic appliances [34].

If loosening occurs prematurely, it may destabilize the prosthetic device, potentially delaying healing or leading to unsatisfactory treatment outcomes [32]. Additionally, TAD failure rates tend to increase in patients with underlying health conditions, such as diabetes or osteoporosis, which impair bone healing and osseointegration [37].

## 4. Combined Orthodontic-prosthetic Treatments: TADs as a Bridging Tool

The application of TADs in combined orthodontic and prosthetic treatments has been extensively explored, showcasing their versatility in addressing complex dental and skeletal issues. Recent studies underscore the importance of interdisciplinary planning when combining orthodontics and prosthetics. In cases where malocclusion or tooth misalignment must be corrected before prosthetic work, TADs provide the necessary anchorage for orthodontic movements, allowing for more precise adjustments without affecting the surrounding teeth [52]. Once the teeth are properly aligned, the prosthetic treatment, whether a crown, bridge, or denture, can be designed to fit seamlessly within the corrected dental arch [53]. This synergy not only enhances aesthetic outcomes but also improves the long-term stability and functionality of the prosthetic device [54].

Takaki et al.[38] conducted a clinical study involving 455 patients and 904 TADs. They observed a high success rate (approximately 90%) across different types of implants, including mini-plates and screws. This case series highlighted the minimal invasiveness and reliability of these devices in various complex orthodontic conditions, including malocclusion and jaw deformities, with low failure rates across different TAD types [38]. Also, Najjar et al.[7] detailed the use of TADs in managing complex orthodontic cases such as Class II and III malocclusions, deep bites, and impacted teeth. The study emphasized the importance of correct TAD placement and maintenance, concluding that these devices significantly reduced treatment time and enhanced the stability of orthodontic movements [7]. A study that was conducted by Capuozzo et al. [19] illustrated how TADs are used to stabilize canines before prosthetic planning, enabling precise repositioning without affecting adjacent teeth. This approach ensures that the final prosthetic device fits seamlessly into the dental arch [19].

These findings are similar to other studies, which demonstrated that TADs combined with aligners are effective in treating impacted canines, where proper tooth positioning is crucial for future prosthetic work [55]. In addition, Iodice et al.[56] demonstrated that TADs are effective in achieving the necessary occlusal adjustments for prosthetic treatments, particularly in patients needing dental implants. Their study emphasized that digital planning with these devices improves the precision of both orthodontic movements and the subsequent placement of implants or other prosthetic devices [56]. Moreover, another study highlighted the importance of integrating TADs in CAD/CAM-guided prosthetic planning, where these devices assist in achieving accurate tooth positioning that complements the final prosthetic outcome [57]. These devices have also been shown to help maintain vertical space and occlusal stability before prosthetic treatments, as Baby et al.[58] documented in their case study on molar intrusion. They used TADs to restore vertical dimension, making it easier to place prosthetic crowns without invasive procedures like crown lengthening [58]. Overall, the interdisciplinary use of TADs in orthodontic and prosthetic treatments demonstrates their versatility in improving clinical outcomes, reducing treatment times, and ensuring that prosthetic devices integrate smoothly into the dental arches.

### 4.1. Treatment Protocols

**Figure-2:**
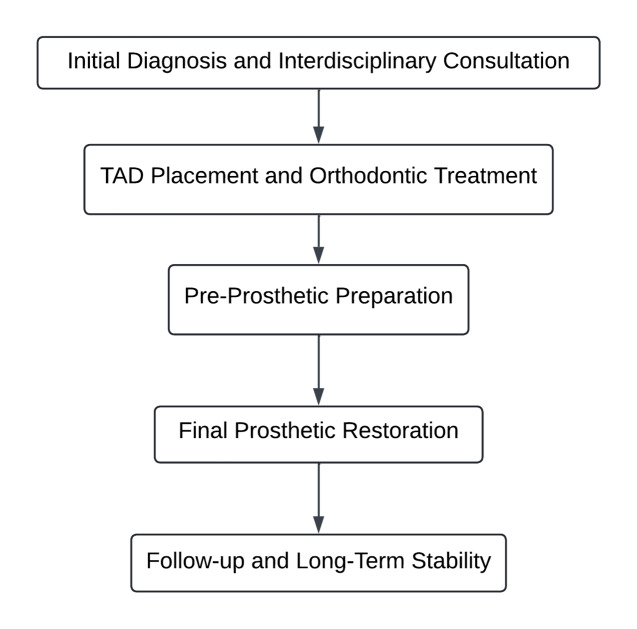
Figure 2. workflow for the integration of TADs in combined orthodontic-prosthetic treatments

The integration of TADs into combined orthodontic-prosthetic treatments requires a well-coordinated approach, with treatment protocols that emphasize interdisciplinary planning. Figure-2 illustrates the approach to treatment planning, including orthodontic corrections followed by prosthetic interventions, with TADs as the anchor [7].

A typical workflow for such combined treatments would include the following steps:

1. Initial diagnosis and interdisciplinary consultation: Begin with comprehensive diagnostics, including CBCT scans, digital impressions, and occlusal analysis. Orthodontists and prosthodontists must collaborate to create a unified treatment plan, determining where and when TADs should be placed based on the final prosthetic goals [19].

2. TAD placement and orthodontic treatment: TADs are placed at strategic locations, typically in the posterior maxilla or mandible, depending on the case's demands. In the case of space closure or tooth intrusion, these devices offer the anchorage necessary for precise movements without disturbing other teeth [59].

3. Pre-prosthetic preparation: Once the orthodontic treatment is completed, TADs may also act as temporary supports for a transitional prosthetic appliance while preparing for permanent implants. During this phase, occlusal adjustments are made to ensure that the dental arches are aligned properly for prosthetic restoration [57].

4. Final prosthetic restoration: After TAD removal, permanent prosthetic devices, such as implants, bridges, or crowns, are placed. This stage focuses on restoring both function and aesthetics, utilizing the orthodontically corrected tooth positions to achieve an optimal occlusal relationship [55].

5. Follow-up and Long-term stability: Regular follow-ups ensure that both the orthodontic corrections and prosthetic restorations maintain stability over time. TAD-related orthodontic movements help improve the integration of prosthetic appliances, reducing the risk of relapse or occlusal dysfunction [12].

### 4.2. Advantages and Limitations

The use of TADs in combined orthodontic-prosthetic treatments provides several key benefits. First, they offer enhanced control over tooth movement, especially in complex cases involving edentulous areas or compromised dental structures [7]. Their ability to provide skeletal anchorage independent of adjacent teeth or soft tissue makes them invaluable where conventional anchorage methods might fail or cause undesirable side effects.[60] TADs also increase treatment versatility by enabling simultaneous orthodontic adjustments and prosthetic preparations, streamlining the treatment process [19].

This reduces the overall time required for both phases, allowing patients to achieve functional and aesthetic outcomes more efficiently [61]. However, there are limitations. One challenge is the technical complexity of placing these devices in areas with insufficient bone density or near vital structures, such as nerves or sinus cavities, where the risk of failure or complications increases [11]. Even with advances in design and placement techniques, complications such as soft tissue irritation, peri-implantitis, or device migration can occur, particularly in patients with poor oral hygiene or health conditions affecting bone healing [62].

Because TADs are temporary, careful coordination with the final prosthetic phase is essential. Failure to remove them at the appropriate time can interfere with the placement of permanent prosthetics or lead to unintended tooth movement [17]. Despite their benefits, potential drawbacks include soft tissue irritation, infection, or failure due to improper placement, particularly in areas of poor bone quality [63]. While traditional anchorage methods avoid surgical risks, they may lack the stability and control required for more complex cases [11].

## 5. Future directions and innovations

As TADs continue to play an integral role in both orthodontic and prosthetic treatments, emerging technologies and biological innovations are set to enhance their effectiveness, precision, and patient outcomes [61]. Recent advancements in 3D printing, digital planning tools, and biomaterial research are transforming how TADs are designed, placed, and integrated into comprehensive treatment plans [64].These innovations are expected to further increase the versatility of TADs, expanding their applications and improving clinical outcomes in various dental and medical fields [65].

### 5.1. New Technologies

One of the most significant innovations in TAD placement and customization is the integration of 3D printing and digital planning tools. Traditionally, the placement of these devices relied heavily on manual techniques and clinical experience, which, while effective, carried a degree of uncertainty, especially in anatomically complex areas [66].

Today, digital workflows, including CBCT imaging and intraoral scanners, allow for precise virtual planning of TAD placement, ensuring optimal positioning for bone density and anatomical structures such as roots, nerves, and sinuses. This precise planning significantly reduces the risk of complications and improves the stability and success of TAD placement [67][68][69].

Moreover, 3D printing technology is now being used to produce customized surgical guides for TAD insertion. These guides are fabricated based on a patient’s unique anatomy, allowing clinicians to insert these devices with exacting precision [70][71]. This level of customization has been shown to enhance the predictability of these procedures, particularly in complex cases involving compromised bone or challenging anatomical structures [64][65].

A recent study demonstrated that 3D-printed guides reduced the margin of error in TAD placement to less than 1mm, thereby minimizing the risk of damaging adjacent roots or structures and increasing the success rate of the treatment [56]. Moreover, robotic-assisted systems are being explored as a future innovation in TAD placement. Similar to their use in dental implant surgeries, robotic systems could further enhance the precision of TAD insertion, particularly in cases where human error could pose a risk [67]. Combined with artificial gene , these systems may eventually automate parts of the diagnostic and placement process, leading to more standardized outcomes and reducing variability between clinicians [68][72].

### 5.2. Biological Considerations

As the use of TADs becomes more widespread, research into biomaterials and surface coatings is focusing on improving osseointegration, the direct anchorage of this method to the bone, which is crucial for long-term stability [73].

While these devices are designed to be temporary, ensuring that they remain securely anchored during the treatment period is essential for their effectiveness. Recent research has explored the use of bioactive coatings, such as hydroxyapatite (HA) and titanium nitride (TiN), to promote better bone integration while reducing the risk of infection and device failure [74][75].

These coatings not only enhance bone-to-TAD contact but also improve the overall biocompatibility of the device. Studies show that these devices coated with bioactive materials exhibit lower failure rates, particularly in areas with low bone density, such as the posterior maxilla [75]. This has been particularly beneficial in patients with compromised bone conditions, such as osteoporosis, where traditional TADs may have had higher failure rates due to poor osseointegration [76]. Furthermore, antibacterial coatings, such as those containing silver nanoparticles, are being researched to prevent peri-implantitis, a common complication caused by bacterial colonization around the TAD site [77][78]. By reducing the incidence of soft tissue infection, these coatings aim to increase the longevity of TAD stability during treatment, especially in patients with compromised oral hygiene or those prone to periodontal issues [78].

### 5.3. Potential for Growth

The future of TADs lies not only in their improving design and placement but also in their potential for broader applications beyond traditional orthodontics and prosthetics [71]. One exciting area of growth is the integration of TADs into fully digital treatment planning systems. With the advancement of digital dentistry, this method could become a standard part of computer-aided orthodontic treatment (CAOT), where the entire treatment workflow, from diagnosis to TAD placement and orthodontic movements, is planned digitally [79]. This integration would allow for a seamless connection between TAD placement and subsequent tooth movement, enabling clinicians to simulate the effects of TAD-supported mechanics before treatment begins [78]. Looking further ahead, TADs could also find applications in maxillofacial surgery, sleep apnea treatments, and craniofacial orthopedic corrections [69].

For instance, in patients requiring orthognathic surgery, these devices could provide critical pre-surgical or post-surgical support, allowing for better alignment of the jaws and teeth. Additionally, TADs could play a role in the treatment of temporomandibular joint disorders (TMD) by providing a stable anchorage for appliances designed to correct mandibular alignment or relieve joint pressure [80]. Finally, the integration of nanotechnology into TAD design holds promise for further enhancing their function. Future TADs may incorporate nano-structured surfaces to promote better cell adhesion and faster healing times, or even drug-delivery systems that release antibiotics or anti-inflammatory agents directly into the surrounding tissue to prevent infection and accelerate recovery [73]. These advancements could not only improve patient outcomes but also expand the use of TADs in a wider range of medical and dental applications.

## Conclusion

TADs have proven to be groundbreaking tools in both orthodontic and prosthetic treatments, offering stable, skeletal anchorage that enhances treatment precision and expands the scope of possible interventions. Throughout this review, we have explored the versatile applications of these devices in complex dental treatments, including their roles in space closure, distalization, and intrusion in orthodontics, as well as their function in providing temporary support for prosthetic appliances in challenging cases, such as edentulous spaces and compromised bone structures. These devices serve as a critical bridge between orthodontics and prosthetics, offering an interdisciplinary approach to patient care that significantly improves functional and aesthetic outcomes.

The role of TADs in combined orthodontic-prosthetic treatments is particularly significant. By offering predictable, non-movable anchorage, this method facilitates more complex tooth movements, which are essential for optimizing the results of prosthetic restorations. This synergy between orthodontics and prosthetics has been well-documented in case studies, where these devices have enabled the precise coordination of tooth alignment and the stabilization of prosthetic devices. These combined treatments, made possible by these devices highlight the increasing importance of interdisciplinary care in dental practice.

TADs represent a significant advancement in the management of complex orthodontic and prosthetic cases, providing improved anchorage control and expanding treatment possibilities. However, while they offer clear advantages, these devices are not without limitations, including risks related to surgical placement and patient-specific factors such as bone quality and oral hygiene.

Despite their widespread success, future research and clinical developments will likely focus on enhancing the design and placement of these devices through emerging technologies. Innovations such as 3D-printed guides, digital planning tools, and robotic-assisted systems are expected to improve placement accuracy and patient outcomes. Furthermore, research into biocompatible coatings and bioactive materials holds the promise of improving osseointegration, reducing complications, and extending the functional life of TADs during treatment. As the field advances, TADs are also poised to play an expanding role in fully digital orthodontic and prosthetic workflows, integrating seamlessly with computer-aided treatment planning and further enhancing their application in complex, interdisciplinary cases. Finally, TADs have already transformed the landscape of dental treatments, particularly in the context of interdisciplinary orthodontic-prosthetic care. With ongoing technological advancements and growing clinical experience, these devices are expected to remain at the forefront of innovation in both orthodontics and prosthetics, offering solutions to increasingly complex clinical challenges. Further research will undoubtedly continue to refine their use, opening up new possibilities for their application in maxillofacial surgery, craniofacial orthopedics, and other fields that rely on precise anchorage systems.

## Conflict of Interest

None.
